# Genomic and Immunologic Markers of Intrinsic Resistance to Pembrolizumab Monotherapy in Microsatellite Instability-High Gastric Cancer: Observations from a Prospective Phase II Study

**DOI:** 10.1055/s-0041-1736237

**Published:** 2022-06-13

**Authors:** Haibo Qiu

**Affiliations:** 1 Department of Gastric Surgery, Sun Yat-sen University Cancer Center, Guangzhou, People's Republic of China

Gastric cancer is one of the most common cancers and a major cause of cancer-related deaths in the world. 1 China is one of the developing countries with the heaviest gastric cancer burden. 2 3 Approximately 50% of all new gastric cancer cases occurred in China. 4 Previous studies reported that 9 to 22% of non-metastatic gastric cancer cases and 3 to 14% of advanced gastric cancer cases have high microsatellite instability (MSI-H). 5 Loss of mismatch repair (MMR) activity is believed to be the main cause of MSI-H. During DNA replication, cells need to use the MMR machinery to detect and correct the small insertions and deletions in DNA sequences, especially the repetitive sequences of microsatellites. Therefore, tumors with loss of MMR activity are characterized by high tumor mutation burden (TMB) and MSI-H. 6 In addition, increase in tumor-infiltrating lymphocytes and programmed death-ligand 1 (PD-L1) expression is common in cancers with MSI-H. 7 These clinicopathologic observations underlie the clinical responsiveness to immune checkpoint inhibitor (ICI) treatment.

Pembrolizumab is a high-affinity monoclonal antibody of programmed cell death-1 (PD-1) designed to prevent interactions between PD-1 and PD-L1. Previous clinical trials have shown that pembrolizumab is a breakthrough drug for metastatic gastric cancer. In the Keynote-059, Keynote-061, and Keynote-062 trials, the overall response rates of MSI-H gastric cancer patients ( *n* = 67) to pembrolizumab monotherapy were 57, 47, and 57%, respectively. 8 In trials with a comparator arm, these outcomes were significantly better than those of control counterparts who received traditional chemotherapy. 9 10 A similar responsiveness was also observed in the treatment for other MSI-H tumors. 11 Based on these findings, pembrolizumab obtained tissue agnostic approval for the treatment of MSI-H tumors. However, irrespective of the kind of MSI-H tumor, the objective response rate (ORR) of pembrolizumab treatment appears to peak at slightly more than 50%, which indicates that about half of the tumors are intrinsically drug resistance. Therefore, there is an urgent need to identify the genomic and immunologic factors that determine the clinical response of pembrolizumab in MSI-H gastric cancer and use these factors to develop strategies to improve the clinical response of pembrolizumab.

In a study recently published in *Cancer Discovery* , titled “Determinants of Response and Intrinsic Resistance to PD-1 Blockade in Microsatellite Instability-High Gastric Cancer,” Kwon et al 12 conducted a phase II clinical trial to gain a deeper understanding of the genomic and immunologic determinants of the pembrolizumab response in MSI-H gastric cancer. A total of 19 advanced MSI-H gastric cancer patients were enrolled in this study. Before receiving the pembrolizumab monotherapy, their tissue biopsy was examined by whole-exome sequencing to infer TMB and genomic variations. After that the patients were categorized by RNA sequencing according to tumor microenvironment components and transcriptional signatures. To examine clonal architecture alterations as a putative response biomarker of pembrolizumab, the authors analyzed early on-treatment multiregion tissue samples by using PhylogicNDT. To explore novel noninvasive biomarkers, they also evaluated the dynamic changes of the immune cell populations in a series of peripheral blood mononuclear cell samples collected continuously.

This study reported that the ORR and disease control rate of the 19-patient cohort were 55.6 and 88.9%, respectively, which are similar to the published data (ORR = 57.1% in Keynote-059 9 ; ORR = 46.7% in Keynote-061 10 ; ORR = 57.1% in Keynote-062 8 ). It is worth noting that the number of patients enrolled in this study are much more than those in the studies mentioned above, and this article also provided a unique group to explore the variable response to PD-1 blockade. In 2019, the burden of indel mutations in MSI-H tumors of engineered mice was found to be strongly associated with the response to PD-1 blockade. 13 In addition, MSI burden (MSIsensor score) was shown to be correlated with the TMB in MSI-H colon cancer patients treated with PD-1/PD-L1 inhibitors. 14 The data from this study supports these findings, and also observed a strong relationship between nonsynonymous single-nucleotide variations and PD-1 response in MSI-H gastric cancer. As we know, MSI-H will cause a large number of frameshifts and single-nucleotide variation mutations eventually. MSI status is the main criterion to distinguish different GC molecular subtypes. Thus, this study tried to explore whether specific recurrent genomic changes are different in MSI-H gastric cancer cases. Although the sample size was small, the authors still identified the genomic characteristics of TCGA genome stable subtypes and mesenchymal subtypes (including the mutations of *CDH1* , *FGFR* , and *RHOA* ) in those patients who responded poorly. Moreover, what deserves our most attention is that there was no association between PD-L1 expression level and clinical outcomes in the MSI-H gastric cancer cohort of this study. Pembrolizumab was approved by the U.S. FDA for the treatment of solid tumors with elevated TMB (>10 Mut/Mb) in 2020. The TMB of all patients enrolled in this study were elevated. As expected, patients with TMB greater than 26 Mut/Mb obtained significant clinical benefits, which indicated that TMB is one of the important factors affecting pembrolizumab response in patients with MSI-H gastric cancer.

To explore the novel characteristics underlying the variable response in a responder enriched population, the authors further examined the on-treatment tissue and plasma collected from the MSI-H gastric cancer patients. Five immune-related features, including the exhaustion status of T cell, the presence of effector T cells in tumor microenvironment, the proportion of T cells and stromal cells, the activation status of T-cell receptor-related signaling, and the status of T-cell receptor repertoire in whole transcriptome analysis, were found to be associated with pembrolizumab response. These findings indicated that the number and functional status of tumor-infiltrating T cells are important factors in determining the antitumor response induced by pembrolizumab. Moreover, the authors found that the inherent high mutation rate of MSI-H gastric cancer may lead to the production of subclonal somatic cells that can evade the host immune response, which may be one of the reasons for the low response to pembrolizumab treatment. Noninvasive predictors are the most attractive treatment prediction tools. However, there is no noninvasive predictor that can be used to predict the response of immunotherapy in clinical practice currently. This study reported that gastric cancer patients with a high percentage of peripheral PD-1 ^+^ CD8 ^+^ T-cells had better effects of pembrolizumab monotherapy.

This study is currently the largest prospective clinical study of MSI-H gastric cancer, but it still has limitations. For example, the number of patients enrolled in this study is relatively small and the patients enrolled are from a single race. Although the published pooled analysis showed that the clinical outcomes for ICIs in non-small cell lung cancer patients from Asia were significantly better than those in white patients, 15 we could not observe the ethnic differences of pembrolizumab response in MSI-H gastric cancer from the present study. Despite the limitations mentioned above, this prospective clinical trial conducted by Kwon et al highlights the response heterogeneity of pembrolizumab monotherapy in MSI-H gastric cancer patients and identified the genomic and immunologic factors associated with the responsiveness of pembrolizumab monotherapy. These findings can promote the development of strategies to enhance responsiveness of immunotherapy in MSI-H gastric cancer.
